# Admission decisions to intensive care units in the context of the major COVID-19 outbreak: local guidance from the COVID-19 Paris-region area

**DOI:** 10.1186/s13054-020-03021-2

**Published:** 2020-06-05

**Authors:** Élie Azoulay, Sadek Beloucif, Bertrand Guidet, Dominique Pateron, Benoît Vivien, Matthieu Le Dorze

**Affiliations:** 1 Médecine Intensive et Réanimation, APHP, Hôpital Saint-Louis, Paris University, 1 Avenue Claude Vellefaux, 75010 Paris, France; 2 Anesthesia and Critical Care Department, Avicenne Hospital, Paris, France; 3 Medecine Intensive et Réanimation Department, Saint-Antoine Hospital, Paris, France; 4 Emergency Department, Saint-Antoine Hospital, Paris, France; 5 Emergency Department, Neckers Hospital, Paris, France; 6 Anesthesia and Critical Care Department, Lariboisiere Hospital, Paris, France

**Keywords:** Decision-making, Triage, Bereavement, Coronavirus, Mechanical ventilation

## Abstract

SARS-CoV-2 has caused a global pandemic unprecedented in size, spread, severity, and mortality. The influx of patients with severe or life-threatening disease means that in some cases, the available medical resources are not sufficient to meet the needs of all patients. Hence, healthcare providers may be forced to make difficult choices about which patients should be referred to the ICU. This document is intended to provide conceptual support to all healthcare teams currently engaged in the frontline management of the COVID-19 pandemic. It aims to assist physicians in the decision-making process for ICU admission and to help them provide uninterrupted and high-quality care.

## Background

This document was written at the request of the Paris-area healthcare authorities in France. This guidance intended to help professionals coordinate patients’ pathways and standardize practices among centers to avoid acting on a first-come first-served basis. These recommendations will evolve as knowledge about COVID-19 increases. Previous statements have provided insights into the clinical and ethical challenges that critical care specialists are facing [1–4]. Moreover, the review from Joebges and Andorno [5] provided a multinational perspective on the ethics guidelines on COVID-19 triage. Alternatives to the first-come first-serve principle have been emphasized [6] by addressing fundamental values such as maximizing the benefits produced by scarce resources, treating people equally, promoting and rewarding instrumental value, and giving priority to the worst off. Patient’s wills, age-related considerations, social considerations (no decision can me made based on person’s wealth or ability to pay), and the specific issues in patients with cognitive impairment have also been highlighted [5]. As discussed by the authors, guidelines also have the potential to reduce the burden on those who need to determine which patient gets access to a scarce resource.

Overall, the context is that of a global pandemic unprecedented in size, spread, severity, and mortality [7]. The influx of patients with severe or life-threatening disease means that in some cases, the available medical resources are not sufficient to meet the needs of all patients [8]. Patients with severe illness may present directly at the ED, deteriorate secondarily after medical ward admission, or be referred to emergency physicians from long-term care units.

In this exceptional context where human, therapeutic, and material resources may be, or soon become, inadequate, overworked practitioners may be forced to make difficult choices about which patients in the emergency department should be referred to the ICU [9].

The ethical principles of distributive justice, non-maleficence, respect for the autonomy and dignity of patients regardless of their degree of vulnerability, and medical data confidentiality are elementary guidelines for managing these patients with severe forms of COVID-19, but also other patients requiring ICU management for non-COVID-related conditions [10].

Emanuel et al. emphasized the role of governments and policymakers to prevent the scarcity of medical resources [6]. However, they formulated six recommendations that shape the development of guidelines that ensure that individual doctors are never tasked with deciding unaided which patients receive life-saving care and which do not. The six specific recommendations for allocating medical resources in the COVID-19 pandemic have been made based on ethical values such as maximizing benefits, treating equally, promoting and rewarding instrumental value, and giving priority to the worst off [6]. These recommendations include to maximize benefits (saving the most lives and at maximizing improvements in individuals’ post-treatment length of life even when it requires to remove a patient from a ventilator or an ICU bed to provide it to others in need); prioritize health workers for testing, PPE, ICU beds, ventilators, therapeutics, and vaccines; do not allocate on a first-come first-served basis; be responsive to evidence; recognize research participation; and apply the same principles to all COVID-19 and non-COVID-19 patients.

This document is intended to provide conceptual support to all healthcare teams currently engaged in the frontline management of the COVID-19 pandemic. It specifically targets physicians whose culture, training, and/or experience may not have prepared them to the reflection underlying the treatment-limitation decision-making process. This approach cannot be a substitute to a contextualized and personalized clinical decision-making that respects patient’s preferences and values, family preferences, and a deliberative and consensual process within the multidisciplinary team. It has two objectives:

- *To assist physicians in the decision-making process for ICU admission*, to avoid unreasonable therapeutic obstinacy. These decisions of withholding and withdrawing life-sustaining therapies may be made upon admission or during the course of the stay.
- *To help physicians continue to provide uninterrupted and high-quality care*, in particular at the end of life, when close collaboration among the team and with the families and other loved ones is crucial. Patients not admitted to intensive care or for whom treatment-limitation decisions have been taken should have access to the full spectrum of expertise available in the hospital (emergency doctors, medical services, and mobile palliative care units), which ensures that acute palliative care provides end-of-life comfort to the patient and relieves the distress of the families.

The elements of reflection relate to both the general case of intensive care patients and the specific case of patients affected by the COVID-19 pandemic. The decision-making process should result in a personalized decision for each individual, be discussed by physicians from all the specialties involved in managing this crisis, and be adapted locally, over time, according to the features of each healthcare organization, material resources, human resources, and feedback from the healthcare personnel involved.

It is essential that these elements also consider the management needs of critical care patients not affected by COVID-19. This re-evaluation is particularly necessary for the most seriously ill and fragile patients, in whom the initial ICU admission was made in a context of uncertainty (admit then re-evaluate rather than do not admit).

The general strategy must be to anticipate these decisions to the extent possible, regardless of the patient’s location (emergency department, standard medical care departments, LTCU, nursing home, etc.), clinical status (with or without signs of severity), and COVID status. In an emergency context, time constraints weigh heavily on the medical decision, and every effort is needed to ensure that despite this burden, the essential duty of compliance with ethical principles is fulfilled.

For any orientation decision, including outside the context of a pandemic, patients, their relatives, and all healthcare personnel must be informed of the extraordinary and personalized nature of the measures taken. The place and role given to relatives in the decision-making process, and the support they receive, may be limited, however, by exceptional circumstances. Finally, these morally and emotionally difficult issues generate anxiety and stress, and support (psychological and/or spiritual) must be offered to everyone, including patients, relatives, and caregivers. We are aware however that pressures regarding ICU admission and discharge may hamper the possibility of an optimal decision-making process that allows every person at stake to reach a consensual decision [7].

## Principles of the ICU admission decision-making process

**Fig. 1:**
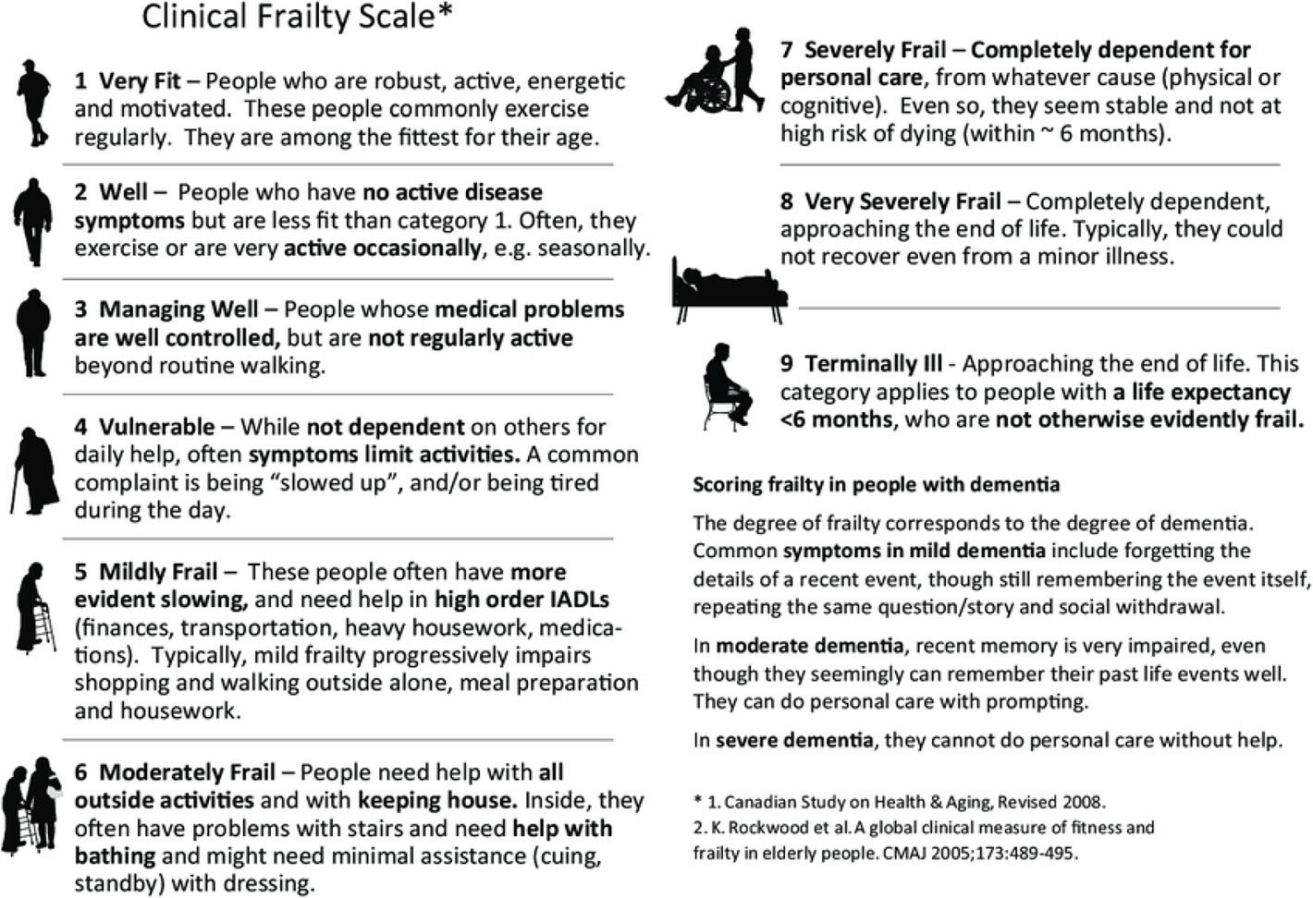
Clinical frailty scale

Despite the health emergency, the collegial procedure required by regulations, legislation, and medical ethics must be followed, with emphasis on the principles listed below [11]:

- Collegiality: the decision remains the responsibility of a single physician, who takes it only after consultation with the healthcare team (the continuity of this collegiality must be organized with at least one other physician and a representative of the non-physician healthcare team)
- Respect for the patient’s wishes and values, expressed directly by the patient via advance directives, indirectly by the patient, or reported by the support person, healthcare surrogate, or next of kin
- Attention to the patient’s previous condition, including at least the following:
  - Frailty assessed using the CFS (Fig. 1) [12]
  - Age (of particular importance for COVID patients)
  - Comorbidities (major vs. stabilized, single vs. multiple)
  - Neurocognitive status (normal, mildly impaired, or severely impaired cognitive functions)
  - Worsening of the patient’s general condition over the last few months
- Attention to current clinical severity, with an assessment of the number of organ failures at the time of decision-making, one of the doctors involved in the latter having examined and spoken with the patient or relatives/support person
  - Respiratory: hypoxemia (> 6 l/min O_2_) or respiratory distress
  - Hemodynamics: systolic blood pressure < 90 mmHg
  - Neurological: Glasgow Coma Scale score < 12
  - Worsening of organ dysfunction
  - Possible use of the SOFA score
- Assessment of the patient’s comfort: pain, anxiety, agitation, dyspnea, congestion, asphyxiation, and isolation
- A full commitment to providing support and care for all, in a way that respects the patient’s dignity

In this context of uncertainty, these decision-making principles apply to both COVID and non-COVID patients. The clinical and contextual data taken into account (such as age, frailty, and comorbidities) are not specific to COVID patients but may have a greater bearing on the nature of the decision taken, depending on the situation.

## Special case of absence of an available bed for a patient meeting criteria for ICU admission

Despite the impressive efforts to extend bed capacities at national, regional, and local levels, this pandemic may lead to a substantial lack of available beds [8]. This situation has been frequently encountered in China, in Italy, and, more recently, in France. This clinical issue arises when a single bed is available but two patients meet criteria for ICU admission. The first logical solution may be to transfer one patient to another ICU with an available bed. Optimal dispatching of patients managed initially by the mobile pre-hospital emergency medical system and the availability of real-time ICU bed counts from Regional Health Agencies and hospitals are paramount.

The second possibility is to optimize the patient’s oxygenation in the hospital or emergency department [13, 14]. This less desirable option may result in sub-optimal treatment and monitoring of the patient and in overcrowding of emergency departments, preventing other patients from accessing them. The ideal solution to this problem is the availability of intermediate structures staffed by qualified personnel and equipped with non-invasive oxygenation devices. However, in the context of a major pandemic, these structures may also eventually reach saturation, leaving the situation unresolved.

The third possibility to avoid loss of chance for the patient requiring ICU admission would be to make a bed available. That includes policies and protocols for early extubation, sometimes with bridge to high flow oxygen therapy or non-invasive ventilation. Opening step-down units dedicated to weaning and non-invasive ventilation appears as a very valuable option. Alternatively, we may discharge a patient already in the ICU (bumping). This solution involves earlier-than-planned extubation of the ICU patient, who is then transferred to an intermediate structure (equipped with high oxygen flow devices and facilities to manage tracheostomized patients). Clinicians may also be keen to implement as early as possible a decision to forgo life-sustaining therapy that includes early extubation with immediate ICU discharge. Again, time constraints weigh heavily here. In a pressured environment with the need for urgent ICU admission, early discharge decisions may also apply to seriously ill patients in whom decisions to forgo life-sustaining therapies have been implemented. It is crucial that these decisions can be made following the best ethical standards, including early introduction of palliative care as well as support for families.

## Residents of LTCUs and nursing homes

Containment and isolation measures must be rigorously enforced for these highly vulnerable patients at high risk of infection. In addition, emergency medical care dispatchers must have easy access to any advance directives and notes written in the medical records. For example, a doctor on call must be reachable 24/7 to participate, if necessary, in a collegial decision to not admit the patient to the ICU. Careful thinking is needed about the optimal modalities for informing the families, who are no longer allowed to visit their frail relatives, since the condition of these patients may deteriorate suddenly.

## In practice

**Fig. 2:**
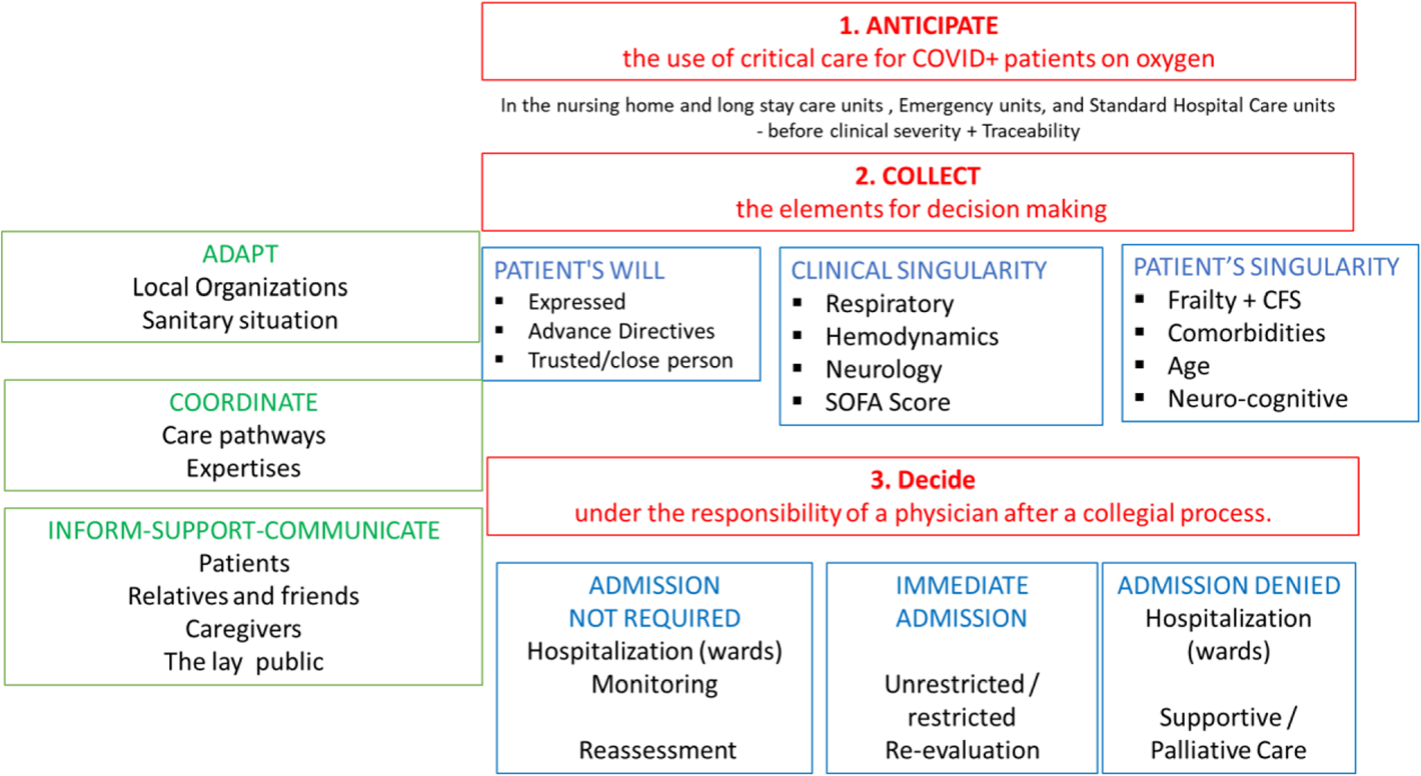
Algorithm for the decision to admit to the ICU a severely hypoxemic COVID-19 patient

The decision-making process involves (see the algorithm below; Fig. 2) the following:

1. Anticipation, as soon as the initial clinical evaluation is carried out, that ICU admission may be required
2. Collection of elements relevant to the clinical analysis of the situation
3. The nature of the decision itself, which may be:
  - Non-admission to critical care:
    i. Because of refusal by the patient and/or family.
    ii. Because the severity criteria do not indicate a need for ICU admission (but instead for another form of continued management, for example, oxygen therapy in a standard care department).
    iii. Because ICU admission would constitute unreasonable obstinacy, defined as treatments or procedures that would not benefit the patient, that would be disproportionate to the expected benefit, and that would have no other purpose than artificially and transiently maintaining life at the expense of suffering for the patient and her/his loved ones and of distress for the staff; ICU admission in this situation may also deprive another patient who has a chance of recovery in the event of ICU admission from receiving ICU care. We therefore consider that it is legitimate not to admit a patient to the ICU when such an admission would constitute unreasonable obstinacy, even when an ICU bed is available.
    iv. Patients not admitted to the ICU receives holistic care as part of an integrated multidisciplinary palliative care program aimed at ensuring the patient’s freedom from suffering and a dignified and peaceful end of life in the presence of her/his loved ones.
  - Admission to critical care:
    i. With periodic re-evaluation, taking into account the response of the organ failures to the treatments used.
    ii. The re-evaluations show whether the patient improves with treatment or, on the contrary, fails to respond, indicating a need to change the goal of treatment from curative to palliative.
4. In every case, any decision, whatever it may be, and its follow-up, must be:

- Recorded and justified in the patient’s medical file, communicated to the caregiver teams, and quickly available in case of emergency.
- Re-evaluated on a regular basis, based on the clinical developments and possible new decision-making elements, as patient survival depends on a response to prolonged supportive treatment, given the absence to date of etiological treatments.
- Clearly and honestly communicated to the patients’ next of kin as the first step of the support process to the families; family conferences and psychological support cells have a valuable role to play.
- Integrate the permanent requirement to limit tensions, both upstream and downstream.

## End-of-life support

The decision to withhold or withdraw acute treatment concerns *therapies*; *care* is always continued [7].

Accompanying patients at the end of life, and their families, remains a priority for healthcare teams everywhere. The patients and families must receive the best support possible from a palliative care team. This support, which takes the form of an acute palliative care approach, is best performed in close collaboration with other medical and palliative care specialists.

The right of every patient to receive analgesia and deep and continuous sedation until death in order to prevent all suffering must be guaranteed [15]. Advance prescriptions supervised by experienced teams must, if necessary, be available to respond to urgent requests for relief.

Efforts must be made to open acute palliative care units designed to meet these needs, as well as to extend critical care capacities [16].

In conclusion, the elements of reflection presented here are proposals rather than formal recommendations. They are obviously evolving and attempt to reconcile the essential ethical imperatives of beneficence and respect for the autonomy and dignity of persons, on the one hand, with efficiency of care, equality, equity, social justice, and distributive justice, on the other. Finally, the documented, objective, and explicit nature of the decision-making elements presented here is intended to be a tool for communication and assistance to patients, relatives, and healthcare teams and to serve as a foundation for the solidarity and trust among all that we must nurture during this ordeal.

## Data Availability

NA, this is a review article.
